# Ball-Milling Enhanced UV Protection Performance of Ca_2_Fe-Sulisobenzone Layered Double Hydroxide Organic Clay

**DOI:** 10.3390/nano14171436

**Published:** 2024-09-02

**Authors:** Márton Szabados, Rebeka Mészáros, Dorina Gabriella Dobó, Zoltán Kónya, Ákos Kukovecz, Pál Sipos

**Affiliations:** 1 Materials and Solution Structure Research Group, Interdisciplinary Excellence Centre, Institute of Chemistry, University of Szeged, Aradi Vértanúk Tere 1, H-6720 Szeged, Hungary; sipos@chem.u-szeged.hu; 2 Department of Molecular and Analytical Chemistry, University of Szeged, Dóm Tér 7–8, H-6720 Szeged, Hungary; 3 Institute of Pharmaceutical Chemistry, University of Szeged, Eötvös Utca 6, H-6720 Szeged, Hungary; m.rebeka92@gmail.com; 4 Institute of Pharmaceutical Technology and Regulatory Affairs, University of Szeged, Eötvös Utca 6, H-6720 Szeged, Hungary; dobo.dorina.gabriella@szte.hu; 5 Department of Applied and Environmental Chemistry, University of Szeged, Rerrich B. Tér 1, H-6720 Szeged, Hungary; konya@chem.u-szeged.hu (Z.K.); kakos@chem.u-szeged.hu (Á.K.); 6 HUN-REN-SZTE Reaction Kinetics and Surface Chemistry Research Group, Rerrich B. Tér 1, H-6720 Szeged, Hungary

**Keywords:** CaFe-hydrocalumite, sulisobenzone intercalation, post-synthetic grinding, vibration and drum mill, optical properties, thermal behaviors

## Abstract

Using a co-precipitation technique, the anionic form of sulisobenzone (benzophenone-4) sunscreen ingredient was incorporated into the interlayer space of CaFe-hydrocalumite for the first time. Using detailed post-synthetic millings of the photoprotective nanocomposite obtained, we aimed to study the mechanochemical effects on complex, hybridized layered double hydroxides (LDHs). Various physicochemical properties of the ground and the intact LDHs were compared by powder X-ray diffractometry, N_2_ adsorption-desorption, UV–Vis diffuse reflectance, infrared and Raman spectroscopy, scanning electron microscopy and thermogravimetric measurements. The data showed significant structural and morphological deformations, surface and textural changes and multifarious thermal behavior. The most interesting development was the change in the optical properties of organic LDHs; the milling significantly improved the UV light blocking ability, especially around 320 nm. Spectroscopic results verified that this could be explained by a modification in interaction between the LDH layers and the sulisobenzone anions, through modulated π–π conjugation and light absorption of benzene rings. In addition to the vibrating mill often used in the laboratory, the photoprotection reinforcement can also be induced by the drum mill grinding system commonly applied in industry.

## 1. Introduction

Layered double hydroxides are long-researched anionic clays of growing interest, which can be found in nature but can also be produced artificially in large quantities and high purity. They are described by the following general formula: [M(II)_1-x_M(III)_x_(OH)_2_]^x+^[A^n−^_x/n_·mH_2_O)]^x−^, where M(II) and M(III) ions are most often Mg^2+^, Ca^2+^, Mn^2+^, Ni^2+^, Co^2+^, Cu^2+^, Zn^2+^ and Al^3+^, Cr^3+^, Fe^3+^, while A^n−^ is the n-charged exchangeable interlamellar anions and x = M(III)/[M(II)+M(III)] [1,2]. The types of organic anions that can be incorporated (intercalated) between their layers are almost infinite; thus, the potential applications of the resulting hybridized layered structures are enormous. A common area of research is in the healthcare sector, where LDHs are used to store, protect, transport and dose drug molecules, taking advantage of their favorable biocompatibility and physiological stability, as well as their easily tunable physicochemical properties (chemical composition, surface charge density, size distribution, shape, thermal and mechanical stability) [3,4]. Recently, LDH nanocomposites have been frequently studied in inflammation and pain reduction [5,6,7], cancer treatment [8,9,10] or even against ageing [11,12,13]. Furthermore, their cosmetic use is a much-researched area, where the rheological properties of these organic clays can be effectively exploited [14,15]. This includes study on the utilization of LDHs obtained by the intercalation of different sunscreen ingredients, such as sulisobenzone, phenylbenzimidazole sulfonic, cinnamic or p-aminobenzoic acid [16,17,18]. These hybrids have been shown to prevent direct contact between sunscreen components and the skin, which usually causes irritation. Furthermore, interactions between the chromophores and the LDH matrix often result in further enhancement of photoprotection and chemical stabilization of the sunscreen molecules. Incorporation of ultraviolet ray absorbents with a benzophenone scaffold, such as sulisobenzone (structural formula in the graphical abstract and in the Supplementary Materials, Figure S1), into (solely) Mg(II)- or Zn(II)-based LDHs has been a topic of research for a long time [19,20,21,22,23,24,25]. Scientists were thus able to make composites that could reduce the oxidation of castor oil [19], serve as additives in the production of transparent UV-resistant ethylene-vinyl alcohol copolymer [22] or enhance the thermal degradation of PVC [23]. And when tested for human dermal fibroblast cells, they have been shown to have negligible cytotoxicity [25].

In the case where the divalent metal ion is calcium, we obtain a separate group of LDHs, noted as hydrocalumites; for all other M(II), we call them hydrotalcite-like materials [26]. Hydrocalumites have a significantly different framework from other LDHs due to the large ionic radius of the Ca(II) ion (compared to the size of the other common M(II) metal ions). Thus, instead of the usual octahedral coordination, a heptahedral one is formed around the Ca(II) ion in the layer, and the value of *x* is fixed at 0.33 [27,28]. This extra coordination site can be occupied by interlayer anions in addition to water molecules, and, therefore, these LDHs have a higher ion-binding capacity compared to the hydrotalcites [29,30]. Among the hydrocalumites, the most common are Ca_2_Al- and Ca_2_Fe-LDHs, which, because of their composition, have excellent biological acceptability. Furthermore, due to the high temperature decomposition of Ca(OH)_2_ (380–400 °C compared to the 100–300 °C of divalent transition metal hydroxides [31]), the Ca-based LDHs also have outstanding and complex, more specific thermal stability [32]. Despite these favorable properties, there are still far fewer examples of the therapeutic use of organo-hydrocalumites than of Mg(II)- or Zn(II)-based hydrotalcites, but this number has started to increase intensively [33,34,35,36,37,38,39,40,41].

Contrary to popular belief, mechanochemistry is far from being limited to physical effects (deformation and fragmentation) in solid materials. Nowadays, mechanical treatments are enjoying a renaissance, as a number of laboratory (attrition, vibration, planetary, roller or drum) ball mills with specialized mechanisms of action are available that can provide sufficiently high energy to break chemical bonds and form new ones. Thus, in the field of LDH synthesis [42,43] and intercalation [40,44] techniques, mechanochemical steps have been successfully used in recent years as low solvent and energy consuming and therefore sustainable developments, and even utilized to successfully produce LDHs with compositions (containing for example Sn(IV) and Ti(IV) ions) that were not possible by other methods [45,46]. In addition, milling as a post-synthetic technique has proven to be an excellent tool to effectively modify many properties of LDHs (specific area and porosity, size, ion-exchange capacity, morphology and thermal behavior) [47,48,49,50,51,52] and to produce mixed metal oxides from LDHs by a specific route instead of calcination [53,54].

Although inexpensive mechanical pre- and post-treatments are often used in the formulation of polymer/clay–drug nanocomposites to achieve better consistency in the end-use [55,56,57], interestingly, to the best of our knowledge, there are virtually zero data on the direct effects of mechanochemical treatments on ready-made hybridized LDHs. Thus, our aim was to create a new, hydrocalumite-based sulisobenzone-LDH photoprotective nanocomposite and to investigate how mechanical treatments can affect the various physicochemical properties, including the light absorption capacity, of such a versatile system.

## 2. Materials and Methods

### 2.1. Materials

Details can be found in the Supplementary Materials.

### 2.2. Preparation and Intercalation Works of the Organic LDHs

Ca_2_Fe-LDHs with or without sulisobenzone (SB) interlayer anion were synthetized by the co-precipitation method of hydrocalumites frequently used in our laboratory [40,52]. At first, the aqueous mixture (7.1 cm^3^) of the 0.3 M Ca(NO_3_)_2_ and 0.15 M Fe(NO_3_)_3_ was prepared and slowly dropped into the also aqueous solution of 3 M NaOH base (20 cm^3^, reaching pH~13). For the encapsulation of the sulisobenzone anions, the acid form of SB was dissolved in the base solution before addition of metal salts. The resulting dispersions were stirred (mechanically, 1000 rpm) at room temperature for 4 days, in closed polypropylene vessels, under N_2_. At the end of syntheses, solids were cleaned by distilled water (150 cm^3^), filtered (0.45 μm pore size), dried and stored in a desiccator at 70 °C, in N_2_.

### 2.3. Mechanochemical Treatments of the Ca_2_Fe-Sulisobenzone Clays

LDH nanocomposites were dry milled in a vibration mill (Retsch MM 400, Retsch GmbH, Haan, Germany); the frequency (12 Hz) and the time (0.5 h) operation parameters were fixed, but the effect of the ball-to-powder mass ratio (BPR) was studied in the range 25 to 600. Grinding balls (25 mm in diameter and ∼60 g in weight) and jars (50 cm^3^ cylindrical internal volume with rounded ends, 37 mm wide and 46 mm long) were made of stainless steel, and the jars were sealed under N_2_ atmosphere. During operation of the mill, the grinding vessel oscillated radially along the horizontal axis, while the ball inside bounced off the rounded ends of the jar. The mill was equipped with two jar receiving units, which allowed each grinding experiment to be carried out at least twice with exactly the same operation parameters. This milling arrangement was suitable for both mild and intensive mechanical treatments and generates specific deformations that are somewhat different from those that would result from circular motion in a commonly applied planetary or roller (drum) ball mill [58].

### 2.4. Methods of Structural Characterization

The obtained solids were probed by powder X-ray diffractometry (XRD), nitrogen gas adsorption–desorption surface and textural analyses, scanning electron microscope combined with spatially-resolved energy dispersive X-ray analysis (SEM-EDX), Raman microscopy, Fourier-transform infrared and UV–Vis diffuse reflectance (DRS) spectroscopies and thermogravimetric tests using a mass spectrometer for the evolved gas investigations (TG-MS), laser diffraction particle size and inductively coupled plasma mass spectrometry (ICP-MS) analyses. Exact measurement parameters are shown in the Supplementary Materials.

## 3. Results and Discussion

### 3.1. Powder X-ray Diffractometry Analysis

**Figure 1:**
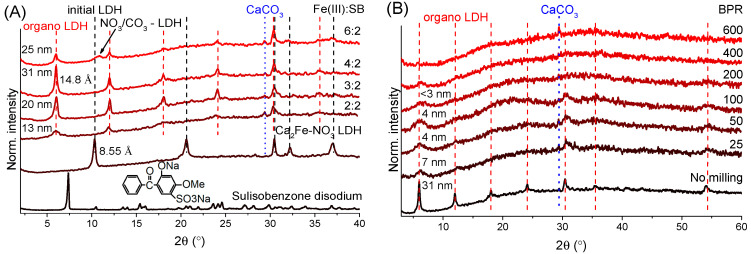
X-ray powder diffraction patterns (**A**) of disodium salt of SB, starting nitrate-containing Ca_2_Fe-LDH and solids prepared with various iron(III):sulisobenzone molar ratios and (**B**) unmilled and milled Ca_2_Fe-sulisobenzone LDH organic clays with increasing ball-to-powder mass ratio (crystal thicknesses and basal distances signed in nm and Å, respectively).

The Fe(III):SB molar ratio used in the synthesis proved to be essential for the production of well-crystallized organic clays. At the same Fe(III) and SB molar amount, the intercalation was successful; the presence of nitrate-containing LDH phase or the sodium salt of sulisobenzone was not detected by XRD tests (Figure 1A). The calculated basal distances (sum of layer thickness and interlayer space) showed a significant increase (from 8.55 to 14.8 Å, the former being characteristic of nitrate-containing Ca_2_Fe-LDHs, JCPDS#48-0065), while periodic arrangement of the newly formed reflections confirmed the preservation of LDH structure. Expansion of the interlayer space (due to the understandably higher space requirement of SB anion compared to the nitrate) was direct evidence of the successful incorporation of SB anions between the layers. In addition to the LDH reflections, only a small amount of CaCO_3_ (JCPDS#47–1743) phase was present in the XRD patterns, which presumably formed during the drying, filtering and storage of the solids.

Theoretically, the maximum amount of SB that can be intercalated was close to the Fe(III) content of LDH (to neutralize excess positive charge in the layers), but, at the 2:4 SB:Fe(III) molar ratio, particles with an average crystal thickness of 31 nm were obtained, which is a significant increase compared to the initial 13 nm value (2:2 SB:Fe(III) ratio, Figure 1A). Presumably due to strong metal–sulfur bonds, some of the SB molecules were coordinated to the LDH surface and thus acted as crystal growth poisons, preventing the LDH layers from stacking, but by reducing the added SB content, the *c*-axis crystallization of LDH hexagons could be increased. Moreover, the optimal 1:2 molar ratio is well understood when considering that both the sulfonic acid and the phenolic groups of SB can be deprotonated during the LDH synthesis. Thus, twice less organic ion was needed to compensate for the positive charge of layers compared to the Fe(III) content. Basal spacing values obtained from the literature of SB-containing Mg_2_Al- and Zn_2_Al-LDHs (13.8 Å) also confirmed that our hybridized LDHs had interlamellar SB molecules in the form of divalent anions [59]. Finally, at a SB:Fe(III) ratio of 2:6, there were no longer enough organic anions to produce only LDH with the larger basal distance, so particles with nitrate and presumably carbonate containing layers could also be formed in small amounts. 

ICP-MS and SEM-EDX analyses of LDHs prepared with different starting Fe(III):SB molar ratios showed that increasing the SB content did not allow the theoretical maximum Fe(III):SB molar ratio of 2:1 to be achieved (Table S1). Even at the most favorable initial molar ratio of 4:2 Fe(III):SB for LDH formation, only a 7:2 Fe(III):SB ratio was measured in the composites. However, infrared and Raman spectroscopy measurements (presented below) showed that no nitrate (from the starting metal salts) or carbonate (from atmospheric CO_2_) ions were present in the interlayer space, suggesting that the residual positive charge of the layers was compensated by hydroxide ions, which were also present in high amounts during the synthesis due to the nature of the co-precipitation method. In any case, LDH composites synthesized with a starting molar ratio of 4:2 Fe(III):SB (thus producing the highest crystallite sizes) were used in the further mechanochemical studies.

Investigations of the effects of grinding intensity and duration on the efficiency of mechanical treatments is very common. However, the ball-to-powder mass ratio is a relatively less studied parameter, although it is also a key aspect, especially in industrial processes, determining the amount of product that can be ground in a cycle. The changes seen in the XRD plots were very similar to our previous results [52]; as the kinetic energy transfer increased, the average size of crystallites decreased rapidly, and samples were characterized by gradual amorphization. Fragmentation of the particles and disordering of the crystal structure resulted in a growing baseline rise, which was largely due to the fluorescence of iron atoms caused by inelastic scattering of X-rays for the unmilled LDHs. At a BPR of 600, the full amorphization of layered structure occurred, no reflections connected to the LDH phase and only the CaCO_3_ particles were visible, which could well be explained by the higher hardness of the calcite (3) compared to the LDH (1.5–2 on the Mohs scale).

### 3.2. Scanning Electron Microscopy and Surface/Textural Probes

**Figure 2:**
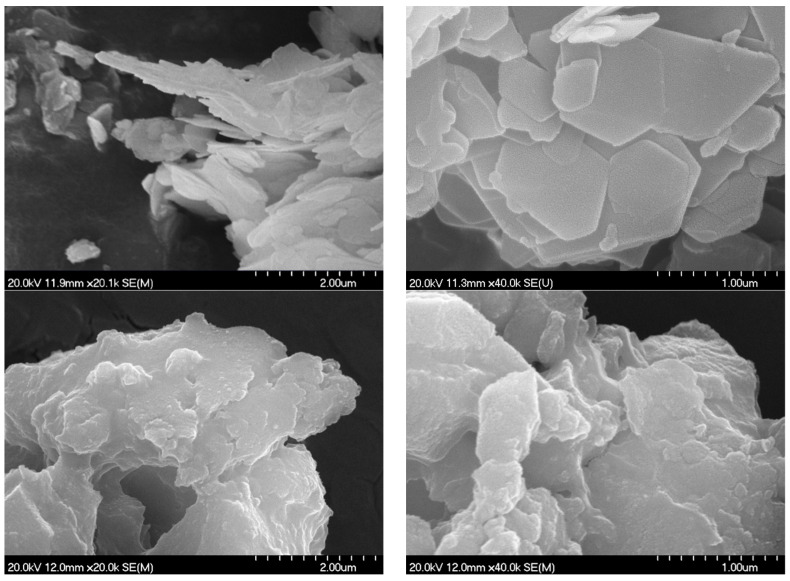
SEM photos of intact (**top**) and milled (BPR: 600) (**bottom**) Ca_2_Fe-SB LDHs.

Electron micrographs proved to be an excellent tool to visualize the amorphization caused by milling (Figure 2). Unground LDH composite nicely exhibited the well-developed hexagonal-shaped particles with distinct contours characteristic of the highly crystallized Ca_2_Fe-LDHs usually containing inorganic anions [52,60]. The average diagonal of hexagons varied between 400 and 1000 nm. As could be guessed from the XRD measurements, the hexagonal morphology disappeared already after grinding with the lowest (25) BPR parameter; only well-aggregated particles with rounded edges were visible (Figure S2). The 600 BPR milling resulted in minimal further change; the particles modulated slightly towards flatter shapes (Figure 2). The spatially resolved elemental mapping showed the uniform distribution of Ca, Fe and S atoms in the starting nanocomposite (Figure S3), indirectly confirming the successful SB intercalation. This was not changed by the grinding process; no segregation of these atoms was detected even after 600 BPR milling (Figure S4).

N_2_ adsorption–desorption analysis of the unground Ca_2_Fe-SB LDH showed a type IV isotherm with an H3 hysteresis loop (Figure S5). This is typical for the well-crystallized LDHs possessing hexagonal, plate-like morphology, with most of the slit-like mesoporous being the voids and space enclosed by the planar particles [49,52,61]. As expected from XRD and SEM results, even grinding at the lowest BPR drastically reduced the average pore size (decreasing the volume of larger pores to a greater extent, Figure S5) as the layered structure was collapsed and the flat hexagonal particles were destroyed. This further resulted in a significant reduction in specific surface area and total pore volume, gradually up to BPR 100 grinding (Table 1). This was considered a turning point, with millings above BPR causing the surface parameters to rise again and settle to a steady state value. A similar trend was described by Pagano [50] and Olszówka et al. [51] for the grinding of MgAl-LDHs. Presumably, in the case of high BPR millings, the number of new surfaces resulting from the intense particle fragmentation could compensate for the reduction of intra-aggregate voids due to the compaction of flat grains. Despite the significant changes in structural, morphological and surface properties, the isotherms of ground samples were relatively similar to the initial composite curves, the milled LDHs retaining their mesoporous character while their pore size distribution is narrowed to the range of 4–10 nm (Figure S5).

### 3.3. UV–Vis DRS Study

**Figure 3:**
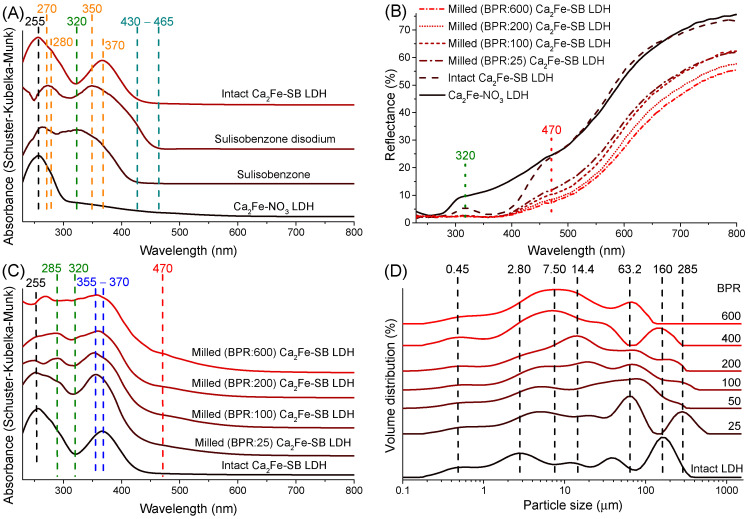
UV–Vis diffuse reflectance spectroscopy analysis of starting nitrate-containing Ca_2_Fe-LDH, SB, disodium salt of SB and unmilled, milled SB-LDHs with increasing ball-to-powder mass ratios ((**A**,**C**)—absorption, (**B**)—reflection spectra). Particle size distribution of unmilled and milled SB-LDH nanocomposites (**D**).

In order to obtain information on the UV–Vis radiation blocking ability of the as-prepared hybridized composites, reflection and absorption spectra were presented and compared to those of the starting nitrate-containing Ca_2_Fe-LDH and sulisobenzone molecules. Interestingly, the spectra of Ca_2_Fe-SB nanocomposite did not show enhanced light absorption between 430 and 465 nm, which was clearly visible for sulisobenzone disodium (Figure 3A). This, based on the optical properties of previous LDH composites [62,63], suggested at first glance that no specific interactions were formed between the organic chromophore and the Ca_2_Fe-LDH matrix. Nevertheless, the interlayer SB anions significantly altered the optical properties of the conventional (nitrate-containing) Ca_2_Fe-LDH. Two new absorption signs (around 280 nm, a shoulder of the 255 nm peak and a new peak at 370 nm) were observed compared to the original one at 255 nm connected to the ligand-to-metal charge transfer. These two absorptions were originally found at 270 and 350 nm in Na_2_SB; hence, their shift attests that some interaction between the π–π conjugated benzene rings -C=O group system and the metal hydroxide layers. Intensity of the absorption of Fe(III) ions (around 410 and 550 nm) was negligible compared to the bands of SB molecules. The spectrum obtained by SB intercalation showed significant light absorption in the UV region, but a decrease was observed around 320 nm (Figure 3A–C), indicating that the light blocking capacity of the as-prepared organic clay was not complete.

From the lowest BPR parameter, the grinding resulted in a significant reduction in the light reflection of LDHs, even to an extent visible to the naked eye. Depending largely on the BPR number used, on average 10–20% less light was reflected from the ground solids than from the unground LDHs between 400 and 800 nm (Figure 3B). As light scattering attributes of the solids are highly dependent on size and size distribution, the decrease in reflectance can be well-paralleled with the observed fragmentation during grinding. According to the laser diffraction size analysis (Figure 3D), increasing BPR value gradually reduced the extent of particles from the initial most common size of 160 down to 8 microns. The relatively rapid rate of fragmentation, particularly focusing on large grains, is well-illustrated by the remarkable decrease in volume-weighted (De Brouckere) mean diameters from an initial value of around 63 to the final 20 microns (Table 1). However, as can be seen from the variation of d(0.1), d(0.5) and d(0.9) particle sizes (Table 1), during the grinding, not only the disintegration of the larger particles but also the aggregation of the smaller ones was observed, moving towards more uniform states in terms of size distribution. Given that this type of particle size analysis does not require any sample preparation, it can provide very reliable results directly on the effect of milling.

At the smallest (25) BPR milling, a slight blue shift of the 370 nm peak and a small increase in light absorption around 285 nm were already visible (Figure 3C). Interestingly, the grinding was able to significantly enhance the UV protection; the gap visible at 320 nm gradually disappeared between 25 and 600 BPR milling. The decrease in particle size, nevertheless, could not fully explain these, as the reflectance differences between the ground solids decreased steadily from 800 to 400 nm (Figure 3B). With increasing BPR values, a weak new peak was observed in the absorption spectra around 470 nm, presumably due to the appearance of iron oxide particles [64]. Similar observations were made in our previous work with increasing the grinding time of kaolinites [65], suggesting that by growing the BPR values, i.e., the energy transferred per unit mass, the LDH layers underwent a small not only layered structural but also some chemical environment transformation. These suggest that several factors may be behind the development of the UV blocking capacity of organo Ca_2_Fe-LDH nanocomposites. In addition to disintegration (collapse) and partial dehydration of the layered framework, it is also conceivable that the altered interaction of chromophores with the inorganic matrix and/or the chemical degradation/transformation of the SB molecules could be responsible for the increased UV absorption. To further investigate the interlayer space and the state of the sulisobenzone anions in LDHs, we continued with infrared and Raman spectroscopy probes.

### 3.4. Infrared and Raman Spectroscopy Measurements

**Figure 4:**
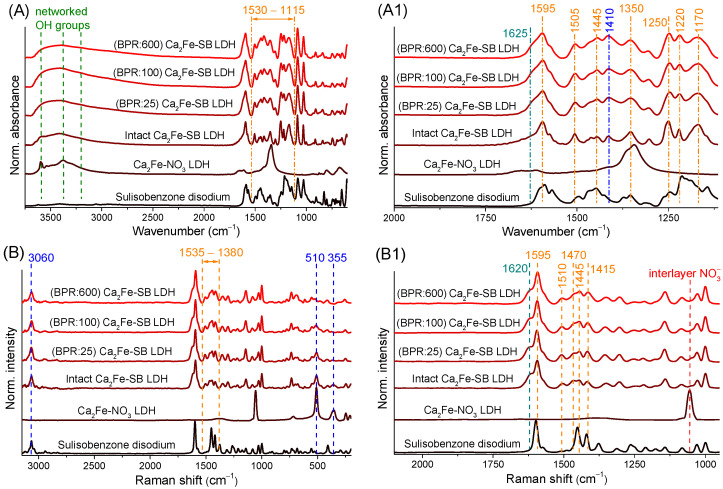
Infrared (**A**,**A1**) and Raman (**B**,**B1**) spectra of starting nitrate-containing Ca_2_Fe-LDH, disodium salt of SB and unmilled, milled Ca_2_Fe-sulisobenzone LDHs with growing BPR.

The infrared spectrum of the organic Ca_2_Fe-LDH showed a completely different vibrational pattern than that of nitrate anion-containing LDH, SB, disodium salt of SB or a mixture of their spectra (Figure S6 and Figure 4A,A1). This was also direct evidence of the incorporation of SB anions into the interlayer space, due to the resulting shifted vibrations caused by strong electrostatic interactions. Mechanical treatments had relatively little effect on the vibrations of organic anions and metal hydroxides; below 1100 cm^−1^ the C-C and aliphatic C-O stretching and C-H deformation modes showed almost no change [66,67]; however, it should be noted that these units did not interact directly with positively charged hydroxide layers. A broad peak between 3000 and 3700 cm^−1^ originated from OH units in the hydrogen bonding network; this part also attested moderate changes, with intensity decreasing and broadening, presumably from grinding induced dehydration and crystal dislocations. The signal broadening was especially higher towards lower wavenumbers, which may indicate a change in intermolecular hydrogen bindings between interlayer anions and layers, and even an increase in the formation of stronger intramolecular bonds. The shoulder at 1625 cm^−1^, the C=O stretching vibration, which becomes slightly more intense with growing BPR, also does not interact directly with the LDH layers but participates in the absorption of UV photons [20,68]. This spectral modulation reflects a change in the immediate environment of the functional group, which can also be linked to the breaking of intermolecular hydrogen bonds and the formation of new intermolecular ones.

Major changes due to grinding were seen in the range 1600/1530–1115 cm^−1^; as the BPR value increased, i.e., the intensity of the mechanochemical treatments, the sign of vibrations strengthened, showing a gradual but not complete fusion (Figure 4A1). Even after 600 BPR milling, the individual vibrations remained separable. Probably, the collapse of the layers and the decay of the interlayer spaces reduced the binding (by ionic and intermolecular forces) of interlamellar anions, leading to freer vibrational states (the formation of new intramolecular hydrogen bonds might also indicate this). This was confirmed by the fact that in this range the vibrations of SB molecule units that interact directly with the layers were well visible. The many overlapping signals made it difficult to identify the bands individually, but this was where C=C benzene rings (around 1595, 1505 and 1445 cm^−1^), -CH_3_ (~1350 cm^−1^), phenolic C-O (~1250 cm^−1^) and sulfonate (~1170 cm^−1^) stretching and aromatic C-H bending (~1220 cm^−1^) vibrations could be found [20,23,24,67,68]. Identification and separation were further complicated by the frequent presence of intense signs of the reversibly surface-adsorbed CO_2_ (~1410 cm^−1^) and calcite (~1480 cm^−1^) peaks in hydrocalumite-type LDHs (from atmospheric CO_2_ content) [40].

Raman spectroscopy measurements (Figure 4B,B1) confirmed the results of the infrared spectra and helped to further narrow the set of organic units that underwent changes during grinding. Vibrations at 510 and 355 cm^−1^ showed a significant attenuation, signaling a high degree of damage to the Ca-O-Fe chains in the LDH framework [40]. Raman spectra attested more clearly that the interlayer nitrate ion (1050 cm^−1^) did not remain in the hybridized Ca_2_Fe-LDHs; meanwhile, the aromatic C-H stretching peak visible at 3060 cm^−1^ and the carbonyl group signal (1620 cm^−1^) showed only very small changes with grinding [69,70]. A similar statement could be made for some of the previously identified functional groups below 1300 cm^−1^, the peaks solely slightly distorted. Both spectroscopic studies showed the broadening of peaks with increasing grinding intensity, which was related to the disintegration of the initial highly crystalline, ordered structure and the formation of a progressively more diverse chemical environment, which could clearly affect the vibrational states of the structural and functional groups. However, between 1535 and 1380 cm^−1^, the bands again showed a strengthening and a mild merging. The majority of these signals (highlighted in Figure 4B1; interestingly, the Raman measurement was less sensitive than the infrared study to milling induced changes for peak 1595 cm^−1^) are all related to aromatic C=C bonds [70,71], which means that the interactions between the layers and the delocalized electrons of benzene rings were mainly altered during the grinding process. This could directly explain the increased light absorption of organic composites by the change in π–π conjugation of SB molecules and the UV absorption of benzene units.

However, it is also worth pointing out that one of the UV absorption maxima of the sulisobenzone (Na-free) molecule is also visible at 320 nm (Figure 3C), and some of the changes seen in the infrared and Raman spectra can be explained by the freer vibrations. These suggest that SB molecules may have been partially released from the layers that disintegrated and even reprotonated by interlamellar and structural water during the mechanochemical impacts. To test the theory, ground LDH particles (BPR 25 and 600) were intensively treated by cyclohexanone (around 40 and 0.04 mg/cm^3^ were the solubility of SB and Na_2_SB in cyclohexanone at 25 °C based on our own tests). In our previous study [60], we observed that Ca_2_Fe-LDHs can be stably stored in cyclohexanone without delamination, mineralization and ion-exchange reactions. Thus, in our present case, solubilization in cyclohexanone allows the removal of SB molecules released from the interlamellar gallery without damaging the layered structure. LDH-cyclohexanone dispersion (10 mg/cm^3^) was magnetically stirred (1000 rpm) for half an hour at room temperature, followed by filtration (by acetone washing), drying and storage steps as described for LDH synthesis. The infrared spectra recorded after washing showed no change; only the vibrations associated with the surface CaCO_3_ formation were enhanced (at 1455, 1405 and 850 cm^−1^), but, in the 1530–1115 cm^−1^ range, the modifications caused by grinding were still well visible (Figure S7A). This clearly shows that the mechanochemical treatments did not allow the sulisobenzone anions to move freely enough to leave the interlayer space in detectable amounts. In addition, any SB molecules that may have been released and reprotonated on the surface could not cause the increase in UV absorbance visible at 320 nm, as the gap in the UV shield was also not registered in the washed samples (Figure S7B).

### 3.5. Thermogravimetric and Evolved Gas Investigations

**Figure 5:**
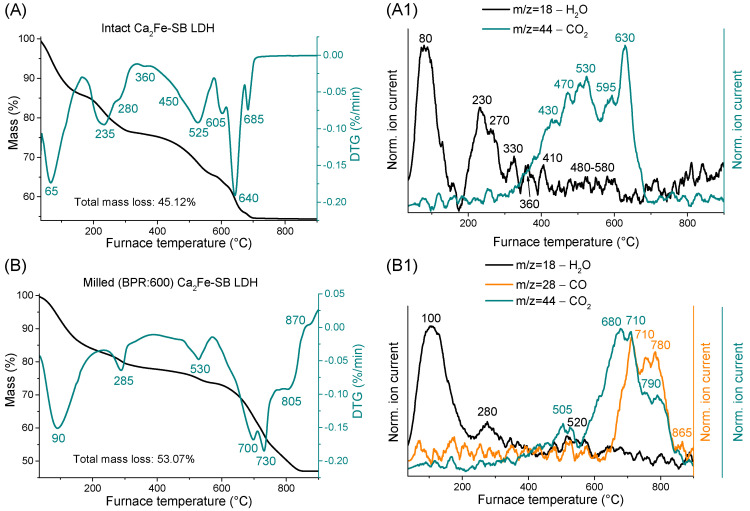
Thermogravimetric, derivative thermogravimetric (DTG) and evolved gas analysis of the starting (**A**,**A1**) and milled (**B**,**B1**) organic clays.

Thermal stability tests of the nitrate-containing LDH and the disodium salt of SB were also performed to gain a more complete understanding of the mechanically changed thermal behavior of the various SB-LDH nanocomposites (Figure S8A–C). TG curves showed well-separated mass losses typical of hydrocalumite systems [2], starting first with the evaporation of physisorbed water on the outer surface. This resulted in mass loss maxima at 125 °C for LDH containing nitrate (Figure S8A) and below 100 °C for organic nanocomposites (Figure 5A,B and Figure S8C). The difference was presumably due to the fact that water molecules were also physisorbed to the SB molecules which were bound to the outer surface of the LDH particles. In the next step, most of the loss was due to the removal of water molecules between layers and, to a lesser extent, to the dehydration of surface Fe-OH units [60]. This is separated by two or three steps, due to the SB intercalation around 235 and 280 °C, compared to the single maximum of 245 °C for nitrate-containing LDH (Figure S8A). Similar observations were made in our previous work where diclofenac and naproxen anions were intercalated into Ca_2_Fe-LDHs [40]. For the ground samples (Figure 5B and Figure S8B), this mass loss again occurred in one step at around 285 °C, presumably due to the disintegration of layers and thus the intense damage of interlayer space. By increasing the BPR parameter, the amount of the removed interlayer water gradually decreased between 160/190 and 330 °C (unground nanocomposite: 9.68% by mass, ground BPR(25): 7.27% and BPR(600): 5.56%). This means that, as a result of grinding, a significant fraction of the water molecules has been released from the interlayer space (and probably from dehydroxylation of surface Fe-OH parts) and evaporated or become surface physisorbed water.

Up to this point, MS measurements had only indicated the removal of water (Figure 5A1,B1 and Figure S8B1,C1), but, from 350 °C, the CO_2_ departure was also visible, suggesting that the dehydration of metal hydroxide layers (around 515 °C for nitrate-containing LDH, Figure S8A) and the onset of decomposition of SB molecules in the nanocomposites were intertwined. Analysis of the disodium salt of SB attested (Figure S8B,B1) that, after the surface water was removed (~75 °C), nothing happens until 330 °C, where the mineralization of molecules commenced with two mass loss maxima (at 365 and 420 °C). It continued up to 800 °C and ended with decarbonization and dehydration of the residual product at 795 °C. LDH layers thermally stabilized the intercalated SB anions (Figure 5A,A1), with their decomposition onset shifted 40/50 °C towards higher temperatures, although a small mass loss at 360 °C was seen, which was attributed to the mineralization of SB molecules bound to the outer surface of LDHs. Interestingly, the interlayer space not only protected the organic molecules but also facilitated their further decomposition; the last CO_2_ removal step in the range studied was significantly reduced from 795 to 685 °C thanks to intercalation. Strong interactions between SB molecules and LDH layers were well-illustrated by the large number of decomposition steps that organic molecules underwent between 400 and 700 °C, while less complicated thermal behavior was observed for the pure Na_2_SB.

Grinding again shifted the mass losses associated with the decomposition of SB molecules towards higher temperatures (Figure 5B,B1 and Figure S8C,C1), while the dehydroxylation temperature of layers did not change significantly (remaining ~520/530 °C). In the ground samples, MS measurements indicated the release of carbon monoxide in addition to the usual water and CO_2_ molecules, suggesting that the mechanical treatments significantly hindered the oxidation of organic moieties in the LDH nanocomposite. This again confirmed that grinding could not help the sulizobenzone anions to break out of the interlamellar gallery. However, between 400 and 900 °C, the rate of mass loss increased significantly due to the mechanochemical treatments (unground nanocomposite: 20.88%, ground BPR(25): 33.22% and BPR(600): 30.80%). This signals that the total oxidation of the organic material was more pronounced, but it should be added that changes due to dehydration of the layers and decomposition of the calcite phase (~690 °C for nitrate-containing LDH, Figure S8A) may have a nuanced effect. The increased tendency to mass loss due to grinding was also reflected in total mass loss values, although it was clear that the complete removal of organic components was never achieved up to 900 °C.

### 3.6. Mechanochemical Treatment of the LDH Nanocomposites in Drum Mill

**Figure 6:**
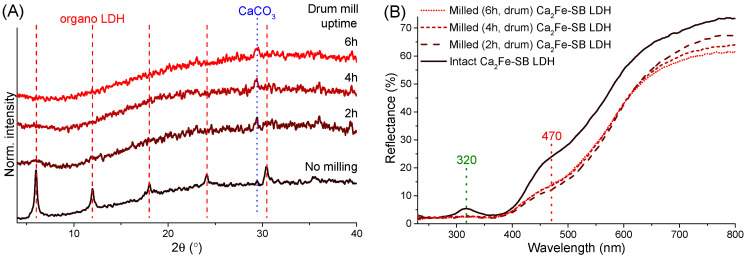
X-ray powder diffraction curves (**A**) and UV–Vis diffuse reflectance spectra (**B**) of the unmilled and milled Ca_2_Fe-sulisobenzone LDHs with increasing drum mill operation time.

Finally, the industrial feasibility of the mechanical production of LDH nanocomposites with enhanced photoprotection was investigated by some exploratory experiments utilizing a drum mill. In contrast to the vibration mill used above, which is mainly applied for short and intensive mechanochemical treatments, drum ball mills are designed for low-energy and continuous grinding processes, which are generally more beneficial for industrial operations. In our drum mill (LE-101, Labor MIM, Esztergom, Hungary), the stainless-steel grinding jar (cylindrical internal volume with rounded ends, 37 mm wide and 46 mm long) rotated horizontally around its own axis, and, for efficient kinetic energy transfer, the, also stainless steel, 12 grinding balls (7 mm in diameter and ∼16.6 g total weight) in the jar moved in waterfall mode [72] at 150 rpm. A BPR of 200 was chosen because at this value the increase in UV protection was clearly visible using the vibration ball mill. The induced structural and optical effects were similar to those previously observed (Figure 1B); after only 2 h of (dry-)grinding, the LDH structure underwent heavy amorphization, the intensity of reflections was significantly reduced, and after 6 h only the calcite phase was identifiable (Figure 6A). DRS analysis indicated a decrease in the amount of reflected light due to grinding; the UV protection defect at 320 nm was eliminated and the light absorption associated with iron oxide particles also increased significantly around 470 nm (Figure 6B). The absorption spectra showed in detail the enhancement of the bands already discussed around 290, 320, 355 and 470 nm. After 2 h of grinding, the hybridized LDH particles showed similar optical behavior as when using the vibration ball mill at 25 BPR, and after 6 h they were similar to 200 BPR (Figure 3C and Figure S9).

## 4. Conclusions

Sulisobenzone anions were successfully intercalated into the interlamellar space of CaFe-hydrocalumites by the conventional co-precipitation technique. By fine-tuning the synthesis recipe, highly crystallized nanocomposite, well-loaded with organic molecules, could be produced. As a result of the intercalation, the thermal stability of the sulisobenzone molecules could be significantly improved and the obtained organic clay had a broad photoprotection potential, but a gap in the UV shield around 320 nm was observed.

Post-synthetic mechanochemical treatments of the hybridized LDHs caused remarkable changes in various physiochemical properties. With the increase in the ball-to-powder mass ratio parameter, layered structure and morphology were intensively degraded, surface and textural parameters were progressively reduced; meanwhile, pore size and particle size distribution became narrower. Thermal and optical properties of the composites were significantly altered by even the mildest grinding used. Thermal decomposition of the interlayer anions had become more complex, and the UV protection had improved.

The change in light absorption capacity was explained by a combination of structural and chemical changes induced by grinding. In addition to the intense particle fragmentation and the partial dehydration of Fe-OH moieties, it was the result of the mechanochemically modified interaction between the positively charged layers and the delocalized electrons of the sulisobenzone molecules. Despite the complexity of the mechanical effects induced, they were achievable not only with a vibration mill designed for high-energy grinding in a laboratory but also with an industrially relevant drum mill with simpler operation and lower power requirements.

## Figures and Tables

**Table 1:** Surface, textural and size distribution properties of the intact and milled Ca_2_Fe-SB LDHs.

BPR	Specific Surface Area (m^2^/g)	Total Pore Volume (cm^3^/g)	Average Pore Diameter (nm)	De Brouckere Mean Diameter (μm)	d(0.1) (μm)	d(0.5) (μm)	d(0.9) (μm)
0	41.7	0.137	14.44	63.88	0.956	14.85	199.5
25	19.8	0.036	7.821	69.94	1.624	21.22	263.6
50	12.3	0.022	7.970	43.11	1.842	21.28	115.0
100	6.92	0.015	8.964	45.41	2.191	17.94	120.4
200	15.3	0.025	6.915	39.59	2.200	15.79	110.6
400	10.7	0.021	8.584	29.90	1.358	8.943	117.7
600	12.6	0.021	7.008	20.08	1.382	9.140	62.07

## Data Availability

All the data are available within the manuscript and Supplementary Materials.

## Supplementary Materials

The following supporting information can be downloaded at: https://www.mdpi.com/article/10.3390/nano14171436/s1, Materials, Methods of structural characterization, Table S1: Composition of the synthesized (SB content was determined based on the amount of sulfur detected) LDH composites; Figure S1: Structural formula of sulisobenzone; Figure S2: SEM photos of the milled (BPR: 25) Ca_2_Fe-sulisobenzone LDHs; Figure S3: SEM elemental maps of the intact Ca_2_Fe-sulisobenzone LDH; Figure S4: SEM elemental maps of the milled (BPR: 600) Ca_2_Fe-sulisobenzone LDH; Figure S5: N_2_ adsorption–desorption isotherms (left) and the corresponding pore size distribution—cumulative pore volume plots (right) of the intact and milled Ca_2_Fe-sulisobenzone LDHs; Figure S6: Infrared spectra of the starting nitrate-containing Ca_2_Fe-LDH, unground Ca_2_Fe-sulisobenzone LDH, disodium salt of the SB and SB molecules; Figure S7: Infrared spectra (A) and UV–Vis diffuse reflectance spectroscopy analysis (B) of the milled Ca_2_Fe-sulisobenzone LDHs after washing with cyclohexanone; Figure S8: Thermogravimetric, derivative thermogravimetric and evolved gas analysis of the starting nitrate-containing Ca_2_Fe-LDH (A), the disodium salt of SB (B,B1) and the milled Ca_2_Fe-sulisobenzone LDH organic clay (C,C1); Figure S9: UV–Vis diffuse absorption spectra of unmilled and milled SB-LDHs with increasing drum mill grinding time.
