# Supplementary material for: Ball-Milling Enhanced UV Protection Performance of Ca2Fe-Sulisobenzone Layered Double Hydroxide Organic Clay
Source: Nanomaterials (Basel). 2024 Sep 2;14(17):1436. doi: 10.3390/nano14171436 (PMC11397241; doi:10.3390/nano14171436)
Supplement: Supplementary file 1 [file nanomaterials-14-01436-s001.zip › nanomaterials-3175938-supplementary.pdf]

Supplementary Materials

Ball-Milling Enhanced UV Protection Performance of Ca_2Fe-Sulisobenzone Layered Double Hydroxide Organic Clay

Márton Szabados[a,b*], Rebeka Mészáros[c], Dorina Gabriella Dobó[d], Zoltán Kónya[e,f], Ákos Kukovecz[e] and Pál Sipos[a,b]

[a]*Materials and Solution Structure Research Group, Interdisciplinary Excellence Centre, Institute of Chemistry, University of Szeged, Aradi vértanúk tere 1, Szeged, H-6720 Hungary*

[b]*Department of Molecular and Analytical Chemistry, University of Szeged, Dóm tér 7-8, Szeged, H-6720 Hungary*

[c]*Institute of Pharmaceutical Chemistry, University of Szeged, Eötvös utca 6, Szeged, H-6720 Hungary*

[d]*Institute of Pharmaceutical Technology and Regulatory Affairs, University of Szeged, Eötvös utca 6, Szeged, H-6720 Hungary*

[e]*Department of Applied and Environmental Chemistry, University of Szeged, Rerrich B. tér 1, Szeged, H-6720 Hungary*

[f]*HUN-REN-SZTE Reaction Kinetics and Surface Chemistry Research Group, Rerrich B. tér 1, Szeged, H-6720 Hungary*

Materials

For the LDH organic clay synthesis, $Ca(NO_3)_2{\times}4H_2O$, $Fe(NO_3)_3{\times}9H_2O$ and sulisobenzone (5-benzoyl-4-hydroxy-2-methoxybenzene-1-sulfonic acid, CAS number: 4065-45-6, Fig. S1) were purchased from the Sigma-Aldrich Company (USA), while the NaOH was obtained from the VWR International (EU). All the solids had high purity (98%+). Disodium salt of the sulisobenzone was prepared by the acid-base reaction of the NaOH aqueous solution and acid form of the sulisobenzone.

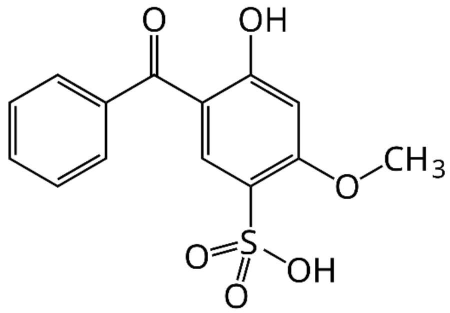

Fig. S1 Structural formula of sulisobenzone.

Correponding author: Márton Szabados
E-mail address: szabados.marton@chem.u-szeged.hu (M. Szabados)

Methods of structural characterization

Powder X-ray diffractograms of the materials were recorded with 2°/min scan speed applying $Cu_{K\alpha}$ (λ = 1.5418 Å) radiation (at 30 kV and 15 mA, by a Rigaku Miniflex II instrument) using Ni foil Kβ filter and scintillation detector in continuous mode with step width of 0.02º 2θ. Reflections were identified applying JCPDS − ICDD (Joint Committee of Powder Diffraction Standards − International Centre for Diffraction Data) database. The coherently scattering domain sizes of LDHs (crystal thicknesses of layers built into each other) were calculated using Scherrer equation at 0.9 shape factor and fitting Gaussian curves on the first reflections.

N_2 adsorption-desorption isotherms were recorded by a Quantachrome Autosorb iQ instrument. Surface of the composites were degassed/cleaned at 150 °C for 2 h under vacuum. The density functional theory method was applied to calculate the average pore sizes and total pore volumes from the adsorption branches, while the Brunauer-Emmett-Teller equation was used to determine the specific surface areas.

Morphologies and spatially-resolved elemental mapping of the solids were probed by scanning electron microscope (Hitachi S-4700) coupled with energy dispersive X-ray spectroscopy (Röntec QX2 spectrometer) at 20 kV acceleration voltage.

The Raman spectra were registered on a Bruker Senterra II Raman microscope at an excitation wavelength of 785 nm and 25 mW laser power level on a sample with spectral shape correction, cosmic spike removal and 600 gr/mm grating. The objective magnification was 50× and the spectra were accumulated from 16 scans for 8 s exposure time.

Fourier-transform infrared spectra of the samples were recorded by a JASCO FT/IR-4700 spectrophotometer accumulating 128 scans at 2 cm^{-1} resolution with ZnSe attenuated total reflectance accessory and deuterated triglycine sulfate detector.

The UV-Vis spectra of the solids were obtained by diffuse reflectance spectroscopy on an Ocean Optics USB4000 spectrometer applying DH-2000-BAL light source. For spectra recording, a remote fiber optic diffuse reflectance accessory with RPH-1 reflection probe holder was used, with the probe tip positioned at 45 degrees with 2 s integration time. To correct for the baseline shift, dark (light source off) and white light (light source on) spectra were recorded with $BaSO_4$ white reference. Based on our previous measurements between 230-250 nm, it can be said that up to 50% reflectance can be reproducibly measured for Ca_2Cr-LDHs, while ~4% for ZnO and less than 0.7% for Mn_4Cr- and Mn_4Fe-LDHs. The

reflectance spectra were converted to absorption curves using the Schuster-Kubelka-Munk equation ($F(R_\infty)=(100-R_\infty)^2/2R_\infty$, where R_∞ was the reflection of infinite layer).

Thermal properties of the samples were revealed by thermogravimetric analysis using a TA Instruments Discovery TGA device connected to a mass spectrometer (MS, Hiden Analytical, HPR-20 EGA). Tests were performed under permanent flow (60 mL/min) of Ar containing 10% oxygen, with a heating rate of 10 °C/min. Electron impact ionisation (70 eV energy) and Faraday cup detectors were applied to identify the ions in the (*m/z*) range of 1 – 300 in full scan mode. The MS transfer line (quartz inert capillary) was heated up to 200 °C and a 20 – 30 mg portion of the solids were placed in the platinum crucibles for the analyses.

Diameters and heterogeneity in particle sizes of the composite grains were mapped by laser diffraction (Malvern Mastersizer 2000, Worcestershire, UK). The solids were dispersed by compressed air, the milled LDHs were without preparation, and the unmilled samples were light pulverization in a mortar before the measurements.

Elemental composition of the nanocomposites was studied applying inductively coupled plasma mass spectrometry (Agilent 7900 ICP-MS, Agilent Technologies, Santa Carla, CA, USA) with ICP multielement solutions (CertiPUR) and yttrium internal standard.

Table S1 Composition of the synthesized (SB content was determined based on the amount of sulfur detected) LDH composites.

	Fe(III):SB molar ratio	Fe(III):SB molar ratio	Fe(III):SB molar ratio	Fe(III):SB molar ratio
Used for synthesis	2:2	3:2	4:2	6:2
Detected in composite	5.4:2	6.2:1	7:2	12:2

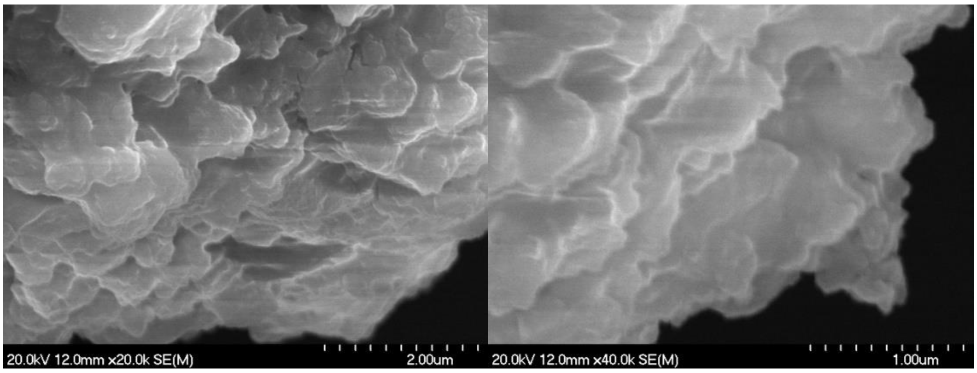

Fig. S2 SEM photos of the milled (BPR: 25) Ca_2Fe-sulisobenzone LDH.

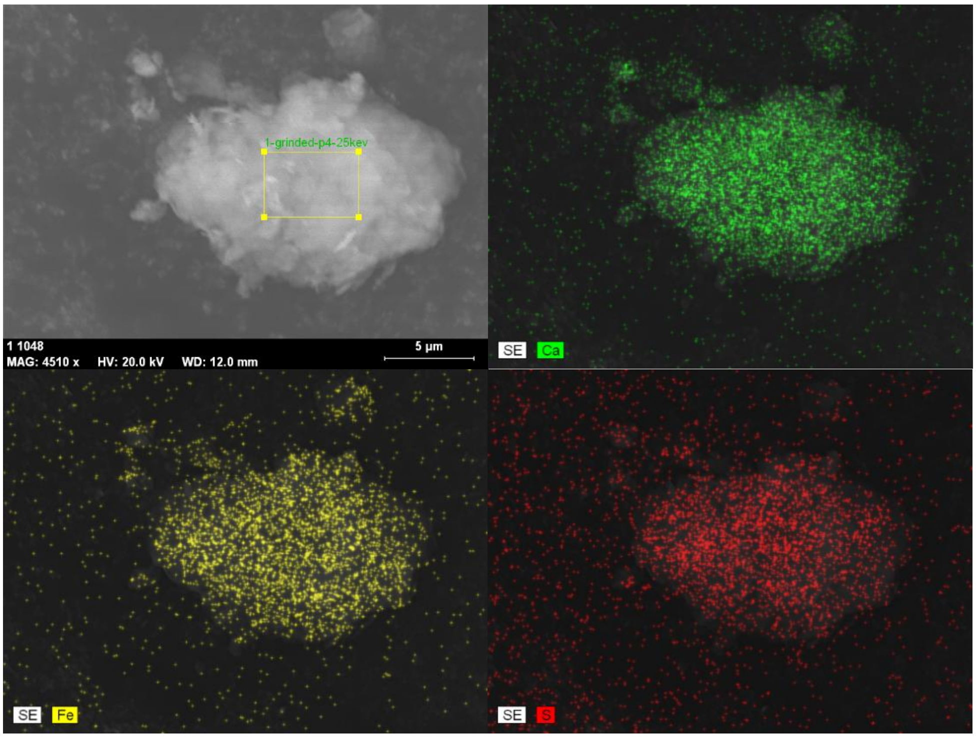

Fig. S3 SEM elemental maps of the intact Ca_2Fe-sulisobenzone LDH.

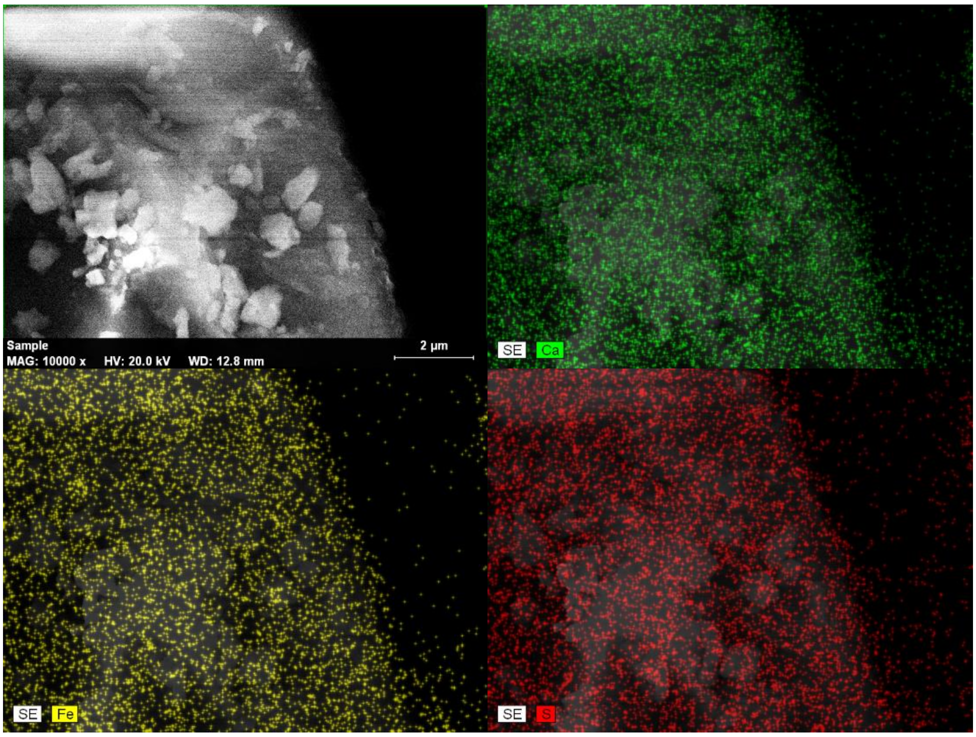

Fig. S4 SEM elemental maps of the milled (BPR: 600) Ca_2Fe-sulisobenzone LDH.

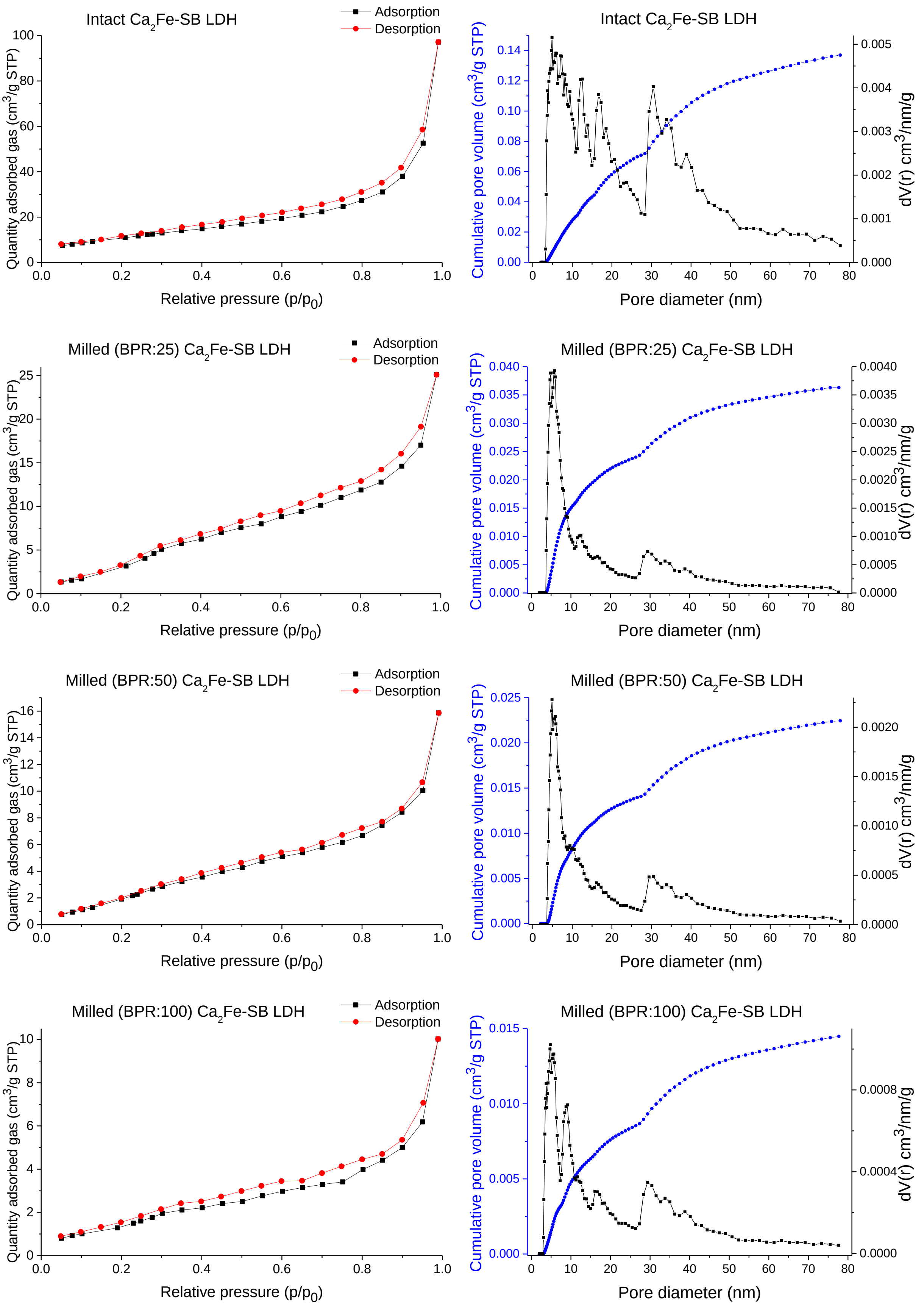
Intact Ca2Fe-SB LDH
Adsorption
Desorption
Quantity adsorbed gas (cm3/g STP)
Relative pressure (p/p0)
Intact Ca2Fe-SB LDH
Cumulative pore volume (cm3/g STP)
dV(r) cm3/nm/g
Pore diameter (nm)
Milled (BPR:25) Ca2Fe-SB LDH
Adsorption
Desorption
Quantity adsorbed gas (cm3/g STP)
Relative pressure (p/p0)
Milled (BPR:25) Ca2Fe-SB LDH
Cumulative pore volume (cm3/g STP)
dV(r) cm3/nm/g
Pore diameter (nm)
Milled (BPR:50) Ca2Fe-SB LDH
Adsorption
Desorption
Quantity adsorbed gas (cm3/g STP)
Relative pressure (p/p0)
Milled (BPR:50) Ca2Fe-SB LDH
Cumulative pore volume (cm3/g STP)
dV(r) cm3/nm/g
Pore diameter (nm)
Milled (BPR:100) Ca2Fe-SB LDH
Adsorption
Desorption
Quantity adsorbed gas (cm3/g STP)
Relative pressure (p/p0)
Milled (BPR:100) Ca2Fe-SB LDH
Cumulative pore volume (cm3/g STP)
dV(r) cm3/nm/g
Pore diameter (nm)

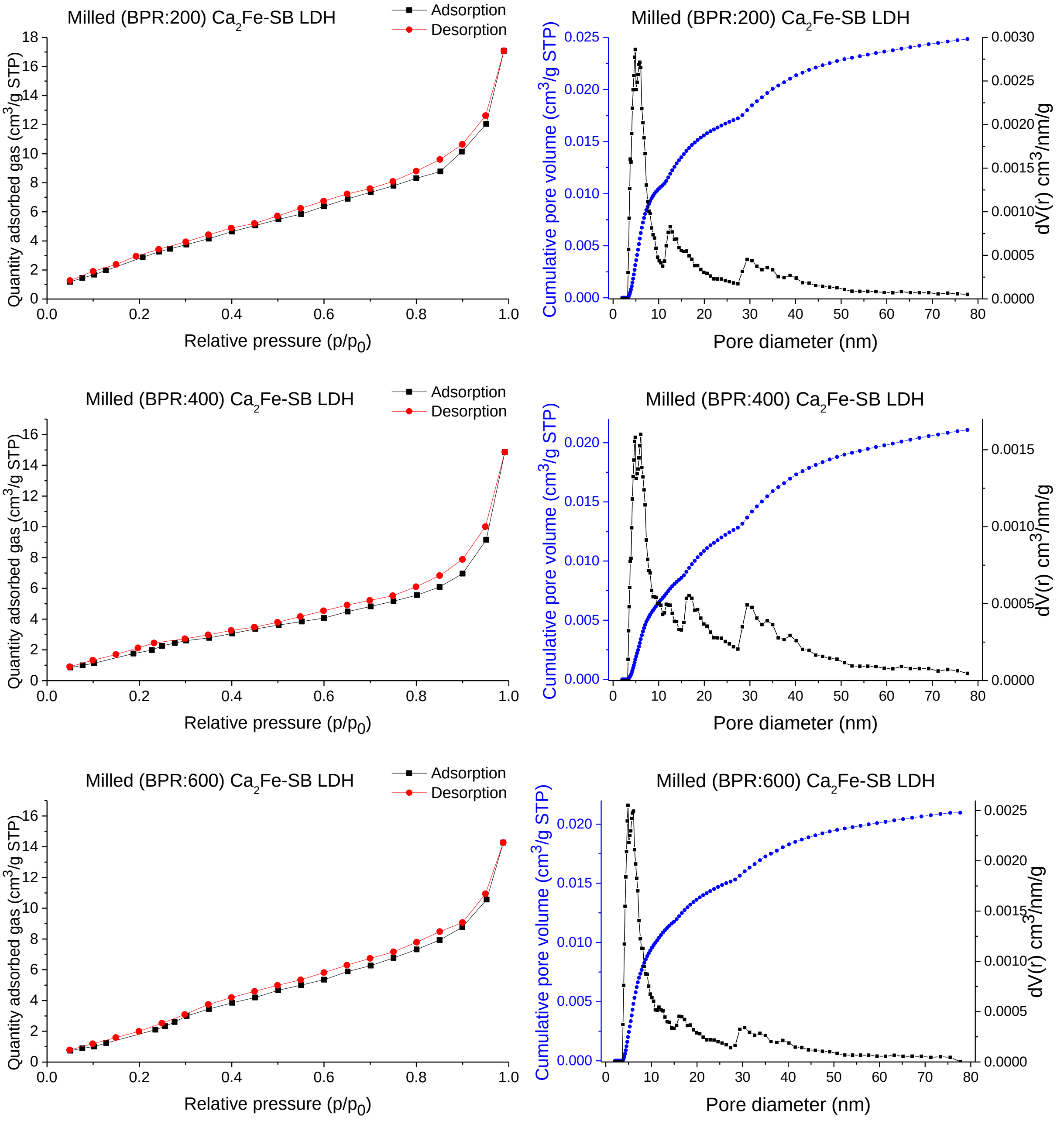

Fig. S5 N_2 adsorption-desorption isotherms (left) and the corresponding pore size distribution - cumulative pore volume plots (right) of the intact and milled Ca_2Fe-sulisobenzone LDHs.

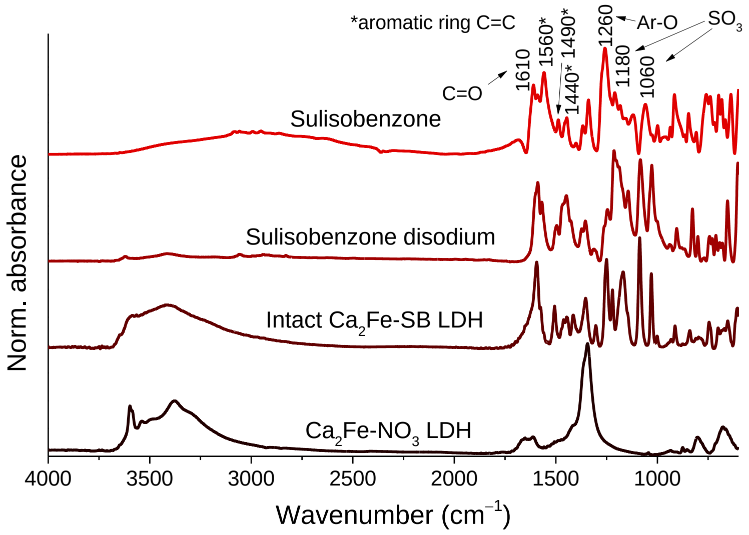

Fig. S6 Infrared spectra of the starting nitrate-containing Ca_2Fe-LDH, unground Ca_2Fe-sulisobenzone LDH, disodium salt of the SB and SB molecules.

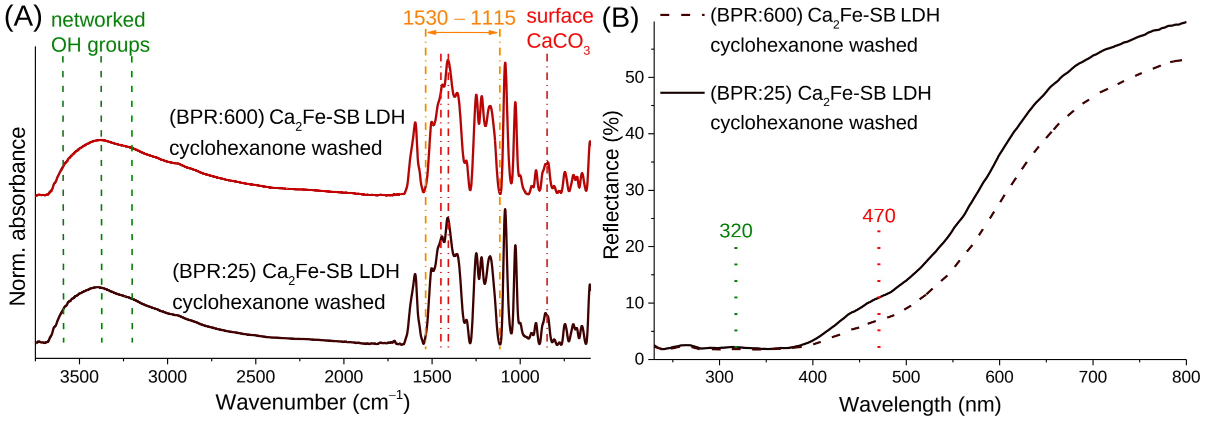

Fig. S7 Infrared spectra (A) and UV–Vis diffuse reflectance spectroscopy analysis (B) of the milled Ca_2Fe-sulisobenzone LDHs after washing with cyclohexanone.

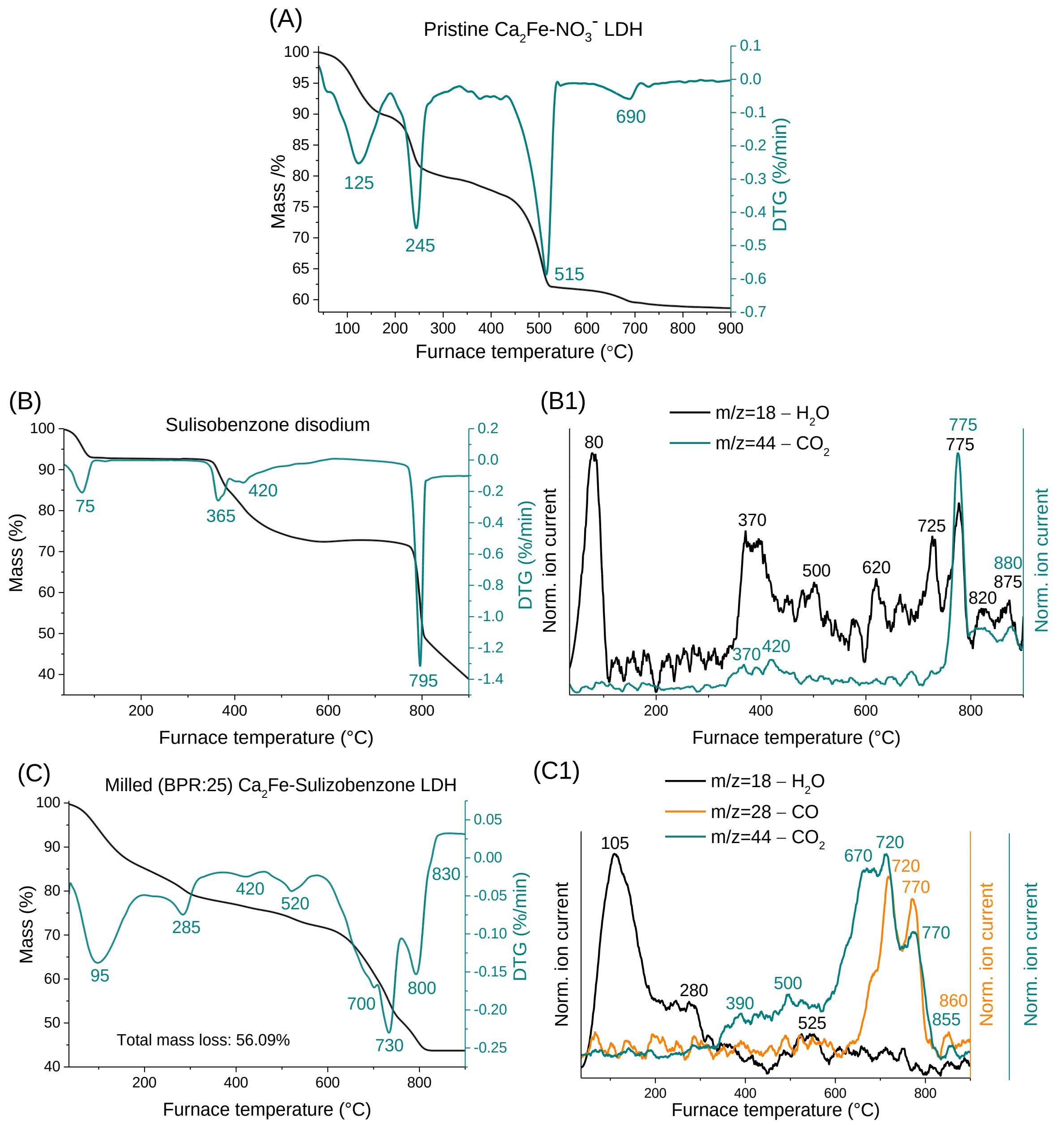

Fig. S8 Thermogravimetric, derivative thermogravimetric and evolved gas analysis of the starting nitrate-containing Ca_2Fe-LDH (A), the disodium salt of SB (B, B1) and the milled Ca_2Fe-sulisobenzone LDH organic clay (C, C1).

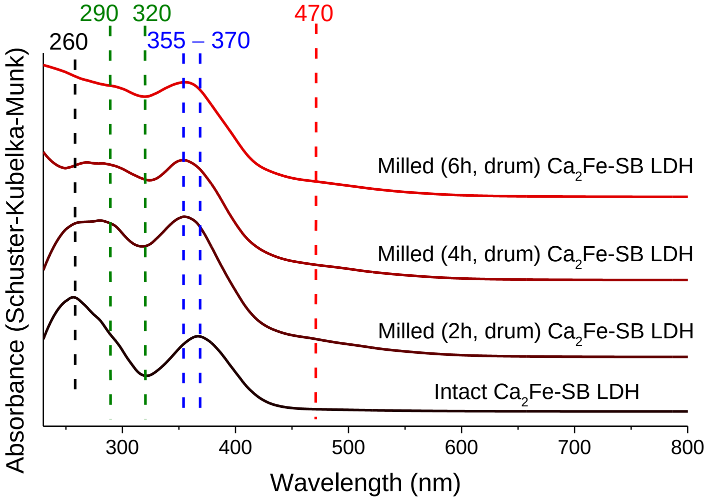

Fig. S9 UV–Vis diffuse absorption spectra of unmilled and milled SB-LDHs with increasing drum mill grinding time.
